# Left versus right approach for middle and lower esophageal squamous cell carcinoma: A propensity score-matched study

**DOI:** 10.3389/fonc.2022.858660

**Published:** 2022-12-13

**Authors:** Xining Zhang, Kang Qi, Weiming Huang, Jingwei Liu, Gang Lin, Jian Li

**Affiliations:** Department of Thoracic Surgery, Peking University Health Science Center, Peking University First Hospital, Beijing, China

**Keywords:** esophageal squamous cell carcinoma, Sweet procedure, Ivor-Lewis procedure, McKeown procedure, lymphadenectomy

## Abstract

**Background:**

Despite superior short-term outcomes, there is considerable debate about the oncological efficacy of the left approach esophagectomy for middle and lower squamous esophageal carcinoma (ESCC). A propensity score-matched retrospective study was conducted to evaluate the left approach’s short- and long-term effects.

**Methods:**

We recorded data from patients with ESCC who underwent curative resection *via* the left or right approach between January 2010 and December 2015. Propensity score matching (PSM) was performed, and maximally selected rank statistics (MSRS) were utilized to determine the appropriate number of lymph nodes to resect during esophagectomy.

**Results:**

One hundred and forty-eight ESCC patients underwent esophagectomy *via* the right approach, and 108 underwent the left approach esophagectomy. After PSM, the left approach esophagectomy showed statistically significant superiority in operative time and time to oral intake, and there was a trend toward a shorter length of hospital stay. Fewer cervical, upper thoracic, and recurrent laryngeal nerve lymph nodes were harvested *via* the left approach than the right approach; the total number of lymph nodes harvested *via* the left and right approaches was similar. Similar long-term survival outcomes were achieved. MSRS suggested that at least 25 lymph nodes are needed to be resected during esophagectomy to improve survival in N0 patients.

**Conclusions:**

The left approach esophagectomy might facilitate postoperative recovery in patients with middle and lower ESCC. With adequate lymphadenectomy, the left approach esophagectomy might achieve similar long-term outcomes for middle and lower ESCC patients.

## 1 Introduction

Esophageal carcinoma is a common malignancy in China, ranked sixth in incidence (285,000) and fourth in mortality (193,000) in 2014 (1). Unlike esophageal adenocarcinoma (the predominant pathological type in the western world), ESCC predominates in East Asia (2). For resectable ESCC, radical resection is the procedure of choice in this era of multi-disciplinary treatment. However, controversies remain regarding the optimal approach for middle and lower ESCC.

The left approach, i.e., a left thoracotomy with or without cervical incision, is common in China, owing (at least in part) to the labor-saving positioning and convenient hilar structure exposure (3, 4). Nevertheless, despite the similar long-term survival outcomes suggested by several studies (3–6), the effectiveness of upper mediastinal lymphadenectomy is debated (7). It appears that this debate between the advocates of left and right approaches was not resolved by a randomized controlled trial (8); there was a critique of the methodology of preoperative evaluation and assignment of adjuvant therapy (9), and there is evidence to suggest the non-inferiority of the Sweet procedure in middle and lower ESCC treatment (10, 11). Therefore, we performed a propensity score matched study to evaluate the effectiveness of the left approach esophagectomy compared to the right approach for middle and lower ESCC.

## 2 Article types

Original research.

## 3 Manuscript

### 3.1 Materials and methods

#### 3.1.1 Study population and groups assignment

Records of patients undergoing curative surgery at Peking University First Hospital between 1 January 2010 and 31 December 2015 were analyzed retrospectively. The ethics committee of Peking University First Hospital approved this study, and consent was acquired for each participant.

Eligibility criteria: 1) age 18 or older; 2) primary squamous cell pathology confirmed; 3) curative surgery undergone; 4) location in the middle or lower esophagus; 5) no distant metastasis suggested before surgery. Exclusion criteria: 1) other histological types; 2) location in the upper esophagus; 3) radical resection not completed, i.e., either the resection was aborted because of intraoperative findings or gross tumor mass remained unresected.

To compare the approaches for middle and lower squamous cell esophageal cancer, we assigned patients into left or right groups based on the procedure; right procedures included Ivor-Lewis and McKeown esophagectomy, and left procedures included thoracic-cervical dual-incision and left thoracic esophagectomy. Because there is evidence suggesting that the greater length of tumor-free esophagus removed with a cervical anastomosis does not result in improved long-term survival (12) (the primary endpoint of our study), the different locations of anastomoses (intrathoracic or cervical) of the same side were included in one group.

#### 3.1.2 Staging and treatment

The preoperative examination included computed tomography (CT) of the chest and upper abdomen, ultrasonography of superficial lymph nodes, cranial magnetic resonance imaging, ultrasonic cardiogram, electrocardiogram, and pulmonary function tests. Positron emission tomography-CT and transesophageal ultrasonography were performed at the surgeon’s discretion. The staging was carried out according to the TNM staging system of the AJCC eighth edition.

**Figure 1:**
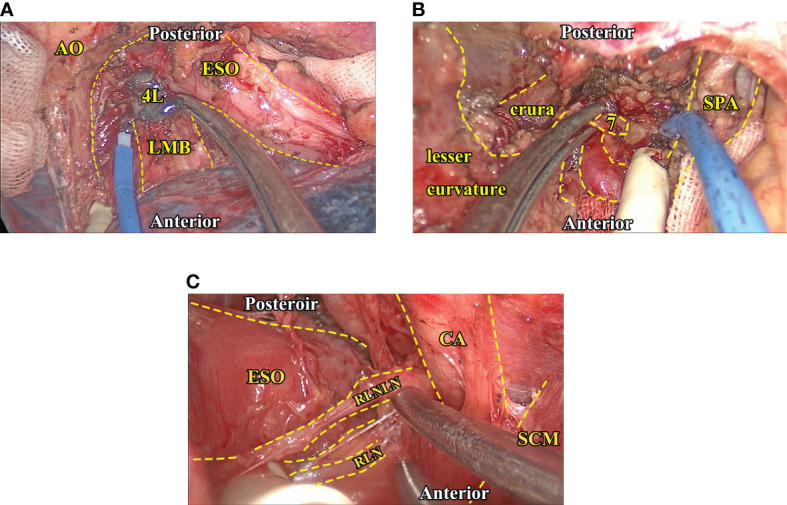
Intraoperative images of critical lymph node exposure and dissection. **(A)**, The exposure and dissection of 4L station (thoracic) lymph nodes. Note that the proper traction of adjacent hilar structures could facilitate exposure of 4L station nodes. **(B)**, The exposure and dissection of seven station (abdominal) lymph nodes after the left gastric vessels are dissected *via* the transhiatal approach. In this region, keeping the stomach empty and anterior traction of it could help expose the celiac structure. **(C)**, The exposure and dissection of recurrent laryngeal nerve lymph nodes (left side), the SCM muscle, and the carotid artery could be gently tracked so the exposure of the peri-esophageal region could be more readily exposed. AO, Aortic artery. CA, Carotid artery. ESO, Esophagus. LMB, Left main bronchus. RLN, Recurrent laryngeal nerve. RLNLN, Recurrent laryngeal nerve lymph nodes. SCM, Sternocleidomastoid muscle. SPA, Splenic artery.

Surgery was conducted *via* either the left (Sweet procedure or left cervicothoracic dual-incision esophagectomy) or right (McKeown or Ivor-Lewis procedure). The left procedure was performed through a left lower intercostal thoracotomy, usually the sixth intercostal space at the mid-axillary line. After the resectability was confirmed by exploration, the middle and lower esophagus was freed, while lymphadenectomy of adjacent lymph nodes was performed in an en bloc fashion. The 4L station lymph node was routinely resected. Then, the abdominal cavity was entered *via* a radial diaphragmatic incision. A series of linear staplers shaped the gastric conduit after adequate length was acquired, and adjacent abdominal lymph nodes were resected in the process. A resection margin more significant than 5 cm was considered safe, and a frozen biopsy was routinely performed to confirm a clear margin. An additional cervical incision would be made if cervical anastomosis was required, and relevant cervical and recurrent laryngeal nerve lymph nodes would be examined and resected in the process if needed. It was worth noting that, as a center that has conducted thoracoscopy-assisted surgery since the early 90s (13), we routinely conduct lymphadenectomy using thoracoscopy, which could greatly facilitate the proper exposure and resection of lymph nodes that lie in the vicinity of the esophagus and the proximal stomach (**Figure 1**). However, although the thoracoscope could greatly facilitate the process, one should acknowledge that the exposure of upper mediastinum lymph nodes would be more readily achieved *via* the right approach. We will address this issue in greater detail in the discussion section. Upon the completion of the anastomosis, a gastric tube was placed with the tip around the diaphragmatic level for decompression and later enteral nutrition.

For the right approach (i.e., the McKeown or the Ivor-Lewis procedure), the esophagus resection and anastomosis placement followed the same principle as the left procedure. Regarding lymphadenectomy, however, the upper mediastinal lymph nodes could be more readily harvested through the right approach; therefore, the major difference between the two approaches lies in the patterns of lymph node resection.

After the procedure, the patients received postoperative care, including prophylactic antibiotics, intravenous patient-controlled anesthesia, and mucolytic treatments. Complete blood counts and biochemistry tests were drawn every other day. Individualized total parenteral nutrition was started on the first postoperative day, and an upper gastrointestinal barium contrast meal was usually conducted on the seventh postoperative day to ensure event-free anastomosis. Following a normal barium meal and blood test results, oral nutrition was immediately given. The patient would be considered eligible for discharge if oral nutrition could be administered without complications and the patient could return to a relatively normal life.

#### 3.1.3 Follow-up and outcome

After discharge, the patient would undergo follow-up every three months for the first two years, then every six months in the third to fifth years. Chest CT, esophageal endoscopy, abdomen and superficial lymph nodes ultrasonography, head magnetic resonance imaging, and serum tumor marker tests were performed at each follow-up. The primary outcomes were overall survival (OS) and recurrence-free survival (RFS). OS was calculated from the date of the surgery to the date of death from any cause, and RFS was calculated from the date of the surgery to the date of disease recurrence. The event-free patient at the final available follow-up date was right-censored at this date in the survival analysis.

#### 3.1.4 PSM, MSRS, and competing risks survival analysis

PSM, a method that limits the bias caused by an existing dataset for nonrandom assignment analysis (14), is used to minimize the inherent bias in a retrospective study. Propensity scores were calculated using logistic regression based on preoperative characteristics, including age, sex, body mass index (BMI), smoking and drinking habits, serum carcinoembryonic antigen and squamous cell carcinoma antigen levels, neoadjuvant therapy, and tumor location. A 1:1 matched cohort was generated by matching patients who underwent the right and left approaches using a caliper width equal to 20% of the standard deviation of propensity scores without replacement. The post-matching balance was tested using the Student’s t-test or Wilcoxon rank-sum test for continuous variables and the chi-square test or Fisher’s exact test for categorical variables.

For the cut-point evaluation, choosing a cut-point that minimizes the p-value of a two-sample test between two groups leads to an increased false error rate. It is necessary to determine whether there is a difference between groups before estimating the cut-point (15). For this purpose, MSRS was used to estimate the cutoff of the number of lymph nodes yielded by radical esophagectomy.

A competing risk is an alternative outcome of equal or more significant clinical importance than the primary outcome that alters the probability of the outcome of interest (16). Competing risk analyses were performed to determine whether the left approach esophagectomy (while offering faster recovery and similar long-term survival) leads to more cervical and mediastinal lymph node recurrence. The sub-hazards of recurrence in the cervical and thoracic lymph nodes and the other regions were calculated using the model developed by Fine and Gray (17), and cumulative incidence functions were plotted.

#### 3.1.5 Statistical analysis

The OS and RFS of patients who underwent the left and right approach esophagectomy were compared using the Kaplan-Meier method in the overall study population and the PSM cohort. A variable would be included in a multivariable Cox regression if the univariable Cox regression suggested significance.

The normality of continuous variables was tested using the Shapiro-Wilk test. Student’s t-test and Wilcoxon rank-sum test were used to compare the normal and skewed distributed continuous variables between groups, respectively. The chi-square test or Fisher’s exact test was performed for categorical variables. For all analyses, p < 0.05 in a two-tailed test was considered statistically significant. All analyses were performed using STATA/MP 15.1 software (StataCorp LLC, College Station, TX, USA), the R Project for Statistical Computing (18), and the R studio.

### 3.2 Results

#### 3.2.1 Characteristics

We identified 256 esophageal squamous cell carcinoma patients who underwent either the left or right approach, including 108 patients with the left approach (13 Sweet procedures and 95 left cervicothoracic dual-incision esophagectomies) and 148 patients with the right approach (40 Ivor-Lewis procedures and 108 McKeown procedures). There were 20 operations in which a radical resection was aborted due to intraoperative findings (e.g., thoracic or abdominal tumor seeding or tumor invasion to vital structures), 8 of those were through the left approach and 12 through the right approach. More patients received neoadjuvant therapy in the right approach group. PSM generated 81 pairs of patients (seven Sweet procedures and 74 left cervicothoracic dual-incision esophagectomies versus 28 Ivor-Lewis procedures and 53 McKeown procedures) whose preoperative characteristics were well balanced (**Table 1**).

**Table 1:** The characteristics of the whole and the propensity score matched cohort.

	Full cohort (N = 256)			Matched cohort (81 pairs)		
	Left approach	Right approach	p-value	Left approach	Right approach	p-value
	N (108)	N (148)		N (81)	N (81)	
Preoperative characteristics						
Age	64.08 ± 9.71	62.41 ± 9.44	0.168	63.11 ± 9.9	65.57 ± 9.56	0.110
Gender			0.161			0.678
Male	94 (87%)	119 (80%)		68 (84%)	66 (81%)	
Female	14 (13%)	29 (20%)		13 (16%)	15 (19%)	
Body mass index	22.45 (20.31-25.71)	23.1 (20.70-25.35)	0.646	21.97 (20.20-24.80)	23.31 (20.82-25.60)	0.135
Smoking habit			0.133			0.423
Yes	57 (53%)	92 (62%)		30 (37%)	35 (43%)	
No	51 (47%)	56 (38%)		51 (63%)	46 (57%)	
Drinking habit			0.871			1.000
Yes	58 (54%)	81 (55%)		31 (38%)	31 (38%)	
No	50 (46%)	67 (45%)		50 (62%)	50 (62%)	
CEA	2.58 (1.53-3.57)	2.56 (1.69-3.82)	0.534	2.63 (1.75-3.60)	1.89 (1.44-3.34)	0.075
SCC	1.1 (0.90-1.78)	1.2 (0.80-1.70)	0.422	1.1 (0.8-1.5)	1.2 (0.9-2.2)	0.113
Neoadjuvant therapy			**0.006**			0.576
No	102 (94%)	123 (83%)		75 (93%)	73 (90%)	
Yes	6 (6%)	25 (17%)		6 (7%)	8 (10%)	
Adjuvant Chemotherapy			0.180			1.000
No	98 (91%)	126 (85%)		74 (91%)	74 (91%)	
Yes	10 (9%)	22 (15%)		7 (9%)	7 (9%)	
Adjuvant Radiotherapy			0.557			0.633
No	92 (85%)	122 (82%)		70 (86%)	72 (89%)	
Yes	16 (15%)	26 (18%)		11 (14%)	9 (11%)	
Pathological results						
Resection margin			1.000			1.000
R0 resection	106 (98%)	144 (97%)		79 (98%)	80 (99%)	
R1 resection	2 (2%)	4 (3%)		2 (2%)	1 (1%)	
Positive lymph node	0 (0-2)	1 (0-2)	0.327	0 (0-2)	0 (0-2)	0.629
Location			**0.000**			1.000
Middle	49 (45%)	110 (74%)		45 (56%)	45 (56%)	
Lower	59 (55%)	38 (26%)		36 (44%)	36 (44%)	
Tumor diameter	3.7 (2.5-4.8)	3.5 (2.5-4.7)	0.778	3.5 (2.3-4.5)	3.5 (2.5-4.9)	0.503
T stage			0.242			0.476
T1	21 (19%)	25 (17%)		16 (20%)	13 (16%)	
T2	28 (26%)	26 (18%)		21 (26%)	17 (21%)	
T3	58 (54%)	96 (65%)		43 (53%)	51 (63%)	
T4a	1 (1%)	1 (1%)		1 (1%)	0 (0%)	
N stage			0.496			0.540
N0	58 (54%)	69 (47%)		44 (54%)	41 (51%)	
N1	30 (28%)	43 (29%)		24 (30%)	23 (28%)	
N2	14 (13%)	29 (20%)		9 (11%)	15 (19%)	
N3	6 (6%)	7 (5%)		4 (5%)	2 (2%)	
TNM Stage			0.475			0.677
I	20 (19%)	18 (12%)		15 (19%)	12 (15%)	
II	41 (38%)	55 (37%)		32 (40%)	31 (38%)	
III	41 (38%)	67 (45%)		30 (37%)	36 (44%)	
IVA	6 (6%)	8 (5%)		4 (5%)	2 (2%)	
Vascular tumor thrombus			0.180			1.000
No	98 (91%)	126 (85%)		72 (89%)	72 (89%)	
Yes	10 (9%)	22 (15%)		9 (11%)	9 (11%)	
Nerve invasion			0.679			0.593
No	86 (80%)	109 (74%)		61 (75%)	58 (72%)	
Yes	26 (24%)	39 (26%)		20 (25%)	23 (28%)	
Differentiation			0.877			0.453
High	12 (11%)	19 (13%)		8 (10%)	13 (16%)	
Moderate	76 (70%)	100 (68%)		59 (73%)	57 (70%)	
Low	20 (19%)	29 (20%)		14 (17%)	11 (14%)	

Variables are presented as mean ± standard deviation, median (first quartile – third quartile) or n (%). CEA, carcinoembryonic antigen; SCC, Squamous cell carcinoma antigen. Bold values denote statistical significance.

#### 3.2.2 Perioperative outcomes

Perioperative outcomes of the whole cohort and the PS-matched cohort are displayed in **Table 2**. The operative time of the left group was significantly shorter than the right approach group in the unmatched and matched groups. After matching, the left group showed a significantly shorter time to oral intake and a trend toward a shorter length of hospital stay. No difference was observed in the severity and incidence of postoperative complications between the groups (**Table 2**). The number of recurrent laryngeal nerves and upper thoracic lymph nodes harvested were less in the left group, and the total lymph node count was similar between the groups in unmatched and matched cohorts (**Table 3**).

**Table 2:** The perioperative outcomes of the whole and the propensity score matched cohort.

	Full cohort (N = 256)			Matched cohort (81 pairs)		
	Left approach	Right approach	p-value	Left approach	Right approach	p-value
	N (108)	N (148)		N (81)	N (81)	
Operation time	417.7 ± 128.28)	499.47 ± 127.36	**0.000**	414.63 ± 130.1	488.59 ± 125.93	**0.000**
Operation bleeding	200 (150-400)	225 (150-400)	0.810	200 (150-350)	300 (175-400)	0.471
Length of hospital stay	11 (10-13.75)	11 (10–14)	0.142	10 (9–13)	12 (10–15)	**0.055**
Time to oral intake	8 (7–10)	9 (7–11)	0.108	8 (7–9)	9 (8-11.5)	**0.019**
Length of ICU stay	0 (0-0)	0 (0-0)	0.972	0 (0-0)	0 (0-0)	0.550
Complication (severity)			0.605			0.719
0	77 (71%)	99 (67%)		58 (72%)	52 (64%)	
1	8 (7%)	19 (13%)		8 (10%)	14 (17%)	
2	14 (13%)	18 (12%)		9 (11%)	8 (10%)	
3	7 (6%)	7 (5%)		4 (5%)	4 (5%)	
4	2 (2%)	5 (3%)		2 (2%)	3 (4%)	
Complication (specific)						
Pulmonary infection	2 (2%)	2 (1%)	1.000	2 (2%)	0 (0%)	0.497
Supraventricular tachycardia	4 (4%)	3 (2%)	0.460	2 (2%)	2 (2%)	1.000
Respiratory failure	1 (1%)	1 (1%)	1.000	1 (1%)	1 (1%)	1.000
CCVI	2 (2%)	3 (2%)	1.000	2 (2%)	2 (2%)	1.000
Chylothorax	4 (4%)	2 (1%)	0.244	3 (4%)	0 (0%)	0.245
Anastomosis leak	6 (6%)	7 (5%)	0.780	2 (2%)	5 (6%)	0.495
Gastroparesis	1 (1%)	2 (1%)	1.000	1 (1%)	1 (1%)	1.000
Wound infection	1 (1%)	1 (1%)	1.000	0 (0%)	1 (1%)	1.000
Recurrent nerve injury	1 (1%)	8 (5%)	0.083	1 (1%)	3 (4%)	0.367
Post-op hemorrhage	1 (1%)	1 (1%)	1.000	1 (1%)	0 (0%)	1.000

Variables are presented as mean ± standard deviation, median (first quartile – third quartile) or n (%). Complications were presented by the Clavien-Dindo classification of surgical complications. ICU, intensive care unit; CCVI, Cerebral-cardiovascular incident. Bold values denote statistical significance.

**Table 3:** The number of lymph nodes harvested of the whole and the propensity score matched cohort.

	Full cohort (N = 256)			Matched cohort (81 pairs)		
	Left approach	Right approach	p-value	Left approach	Right approach	p-value
	N (108)	N (148)		N (81)	N (81)	
Lymph node harvested (total)	23 (19-30)	25 (20-33)	0.173	22 (18.5-29.5)	25 (21-30.5)	0.095
Lymph node harvested (neck)	0 (0-0)	0 (0-0)	0.089	0 (0-0)	0 (0-0)	0.118
Lymph node harvested (RLN)	0 (0-0)	0 (0-3)	**0.000**	0 (0-0)	2 (0-3)	**0.000**
Lymph node harvested (thorax)	13 (9–18)	15 (10-19.8)	**0.034**	13 (9-17.5)	15 (10–20)	**0.029**
Upper thorax	0 (0-0)	1.5 (0-5.8)	**0.000**	0 (0-0)	1 (0-5)	**0.000**
Middle and lower thorax	13 (9-17.8)	12 (8-16)	0.208	13 (9-17)	13 (8-16)	0.741
Lymph node harvested (abdomen)	10 (5–14)	9 (5-13.8)	0.372	10 (5–14)	8 (5–13)	0.396

RLN, recurrent laryngeal nerve. Bold values denote statistical significance.

**Figure 2:**
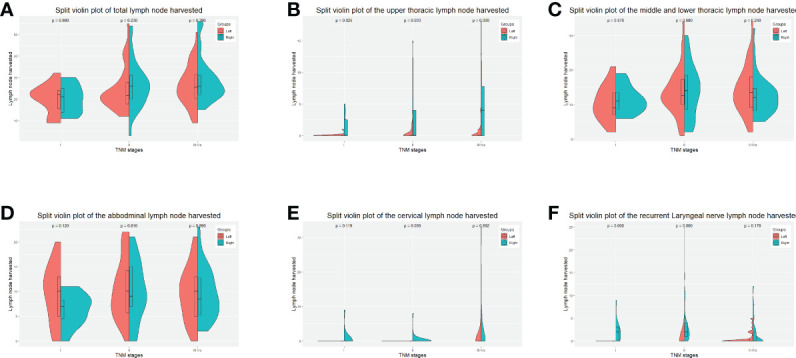
Split-violin plots of amount of lymph nodes resected by the Sweet and the right approaches in the PS-matched cohort. **(A)**, the total amount of lymph nodes resected. **(B)**, the amount of the upper thoracic lymph nodes resected. **(C)**, the amount of the middle and lower thoracic lymph nodes resected. **(D)**, the number of abdominal lymph nodes resected. **(E)**, the number of cervical lymph nodes resected. **(F)**, the amount of recurrent laryngeal nerve lymph nodes resected. PS, propensity score.

We further investigated the lymph node yield in a stratified fashion. The number of lymph nodes harvested by the left approach was less than that of the right approach in upper fields (cervical lymph nodes in stage II p = 0.039, recurrent laryngeal nerve lymph nodes in stage I p < 0.001, stage II p < 0.001, upper thoracic lymph nodes in stage I p = 0.025, stage II p = 0.033, stage III-Iva p < 0.001), the number of lymph nodes harvested *via* the left approach was not significantly less than the right approach in the lower fields (i.e., middle and lower thoracic and abdominal) in any stage (**Figure 2**).

**Figure 3:**
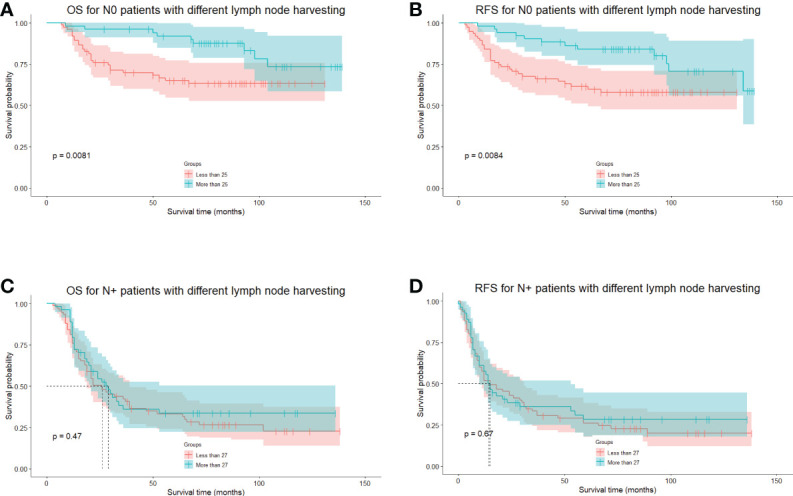
The long-term survival of N-negative and N-positive patients grouped by cutoff points calculated using the MSRS. **(A)**, The OS of N-negative patients divided by the cutoff points of 25 lymph nodes. **(B)**, The RFS of N-negative patients divided by the cutoff points of 25 lymph nodes. **(C)**, The OS of N-positive patients divided by the cutoff points of 27 lymph nodes. **(D)**, The RFS of N-positive patients, divided by the cutoff points of 27 lymph nodes. MSRS, maximally selected rank statistics. OS, overall survival. RFS, recurrence-free survival.

We also investigated the minimal number of lymph node resections for a significant survival benefit using MSRS. The minimum number of lymph nodes harvested during the curative procedure was 25 and 27 for N-negative and N-positive patients. However, no significance was detected between the number of lymph nodes harvested and survival in N-positive patients (**Figure 3**).

#### 3.2.3 Survival outcomes

**Figure 4:**
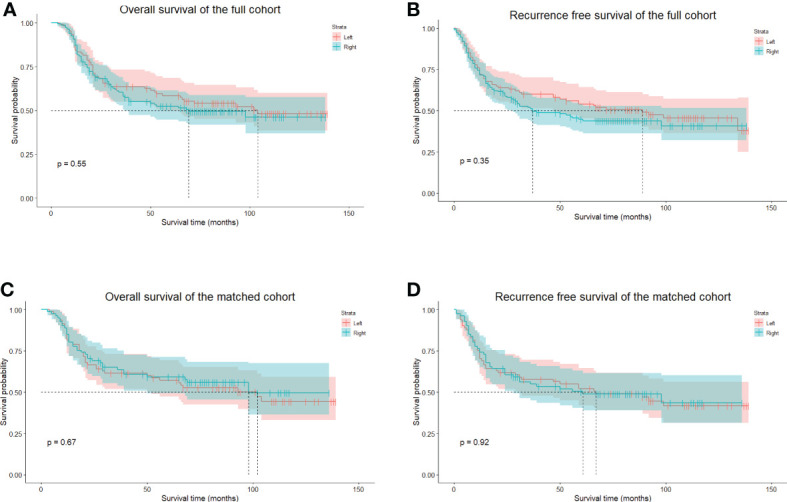
The long-term survival of unmatched and PS-matched cohorts. **(A)**, The OS of unmatched patients in the left and right groups. **(B)**, the RFS of unmatched patients in the left and the right groups. **(C)**, The OS of matched patients in the left and right groups. **(D)**, the RFS of matched patients in the left and the right groups. PS, propensity score. OS, overall survival. RFS, recurrence-free survival.

**Figure 5:**
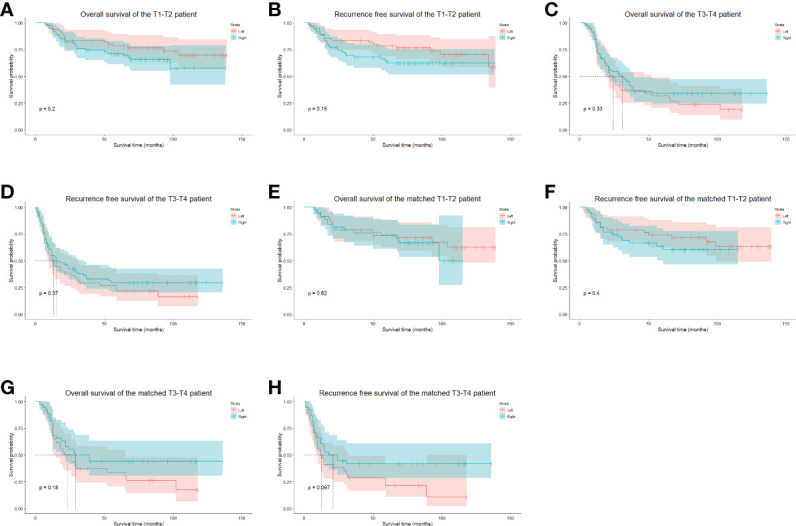
The stratified long-term survival of unmatched and PS-matched cohorts. **(A)**, The OS of unmatched stage T1-2 patients. **(B)**, The RFS of unmatched stage T1-2 patients. **(C)**, The OS of unmatched stage T3-4 patients. **(D)**, The RFS of unmatched stage T3-4 patients. **(E)**, The OS of matched stage T1-2 patients. **(F)**, The RFS of matched stage T1-2 patients. **(G)**, The OS of matched stage T3-4 patients. **(H)**, The RFS of matched stage T3-4 patients. OS, overall survival. RFS, recurrence-free survival.

The median follow-up period was 47.5 months (range 3–139 months), the 5-year OS was 55.02%, and the 5-year RFS was 50.01%. In the whole cohort, the 5-year OS of the left group was 58.37%, and that of the right approach group was 52.41% (p = 0.546) (**Figure 4A**). The 5-year RFSs of the left and right approach groups were 53.93% and 45.00%, respectively (p = 0.354) (**Figure 4B**). After PS matching, the 5-year OSs of the left and right approach groups were 57.20% and 59.31%, respectively (p = 0.669) (**Figure 4C**), and the 5-year RFSs were 52.11% and 50.46%, respectively (p = 0.922) (**Figure 4D**). We also investigated OS and RFS in both groups in unmatched and matched cohorts in a staged manner; no statistically significant difference was found in any stratum (**Figure 5**). In the matched cohort, for patients receiving neoadjuvant therapies, there was no significant difference between the left and the right approaches in terms of 5-year OSs (50.00% vs. 50.00%, p = 0.852) and 5-year RFSs (50.0% vs. 33.3% p = 0.748).

**Figure 6:**
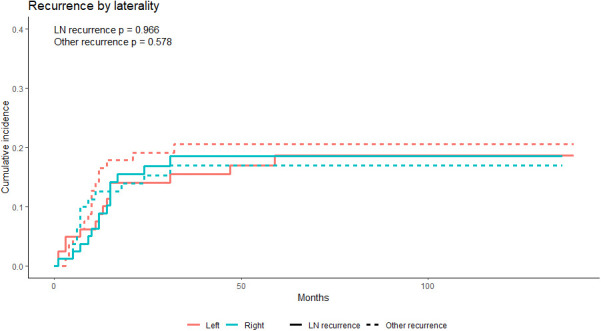
The cumulative incidence functions of the PS-matched left and right groups; neither the recurrence rate of the cervical and mediastinal lymph nodes region nor the other region showed a statistically significant difference. LN, lymph nodes. PS, propensity score.

For competing risks, cumulative incidence functions showed no significant difference between the left and right groups in regional (i.e., cervical and mediastinal lymph node) recurrence and recurrence of other regions (**Figure 6**). In the multivariate competing risks analysis, the left approach esophagectomy showed non-inferiority in the cervical and mediastinal region recurrence rate in the matched cohort (**Table 4**).

**Table 4:** The sub-hazards of the cervical and mediastinal recurrence, competing with recurrence of other sites.

Variables	Sub-Hazards	p-value	95% Confidence interval	
Left or right approach	1.015	0.971	0.444	2.322
Neoadjuvant therapy	3.115	0.125	0.728	13.322
Operative bleeding	1.001	0.282	0.999	1.004
Length of Stay	1.111	0.108	0.977	1.262
Operative time	0.750	**0.006**	0.612	0.919
Adjuvant radiotherapy	11.391	**0.000**	5.120	25.344
Amount of metastasized LN	0.984	0.898	0.765	1.265
Amount of resected cervical LN	1.015	0.712	0.937	1.100
TNM stage	2.099	0.169	0.730	6.037
Carcinothrombosis	2.419	0.260	0.521	11.235
Nerve invasion	2.468	0.072	0.922	6.609

LN, lymph node. Bold values denote statistical significance.

In the multivariate Cox regression analysis, postoperative chemotherapy, pathologic nerve invasion, and a lower BMI were associated with a worse OS. Elevated serum carcinoembryonic antigen, postoperative chemotherapy, pathologic nerve invasion, and a lower BMI with a worse RFS. Neither the extent of lymphadenectomy nor the laterality of the approach had a significant impact on OS and RFS (**Table 5**).

**Table 5:** Multivariate Cox-regression analysis.

Variables	Hazard ratio	p	95% Confidence interval	
Overall survival				
Body mass index	0.923	**0.006**	0.871	0.977
Adjuvant radiotherapy	2.259	**0.001**	1.416	3.603
Tumor diameter	1.126	0.052	0.999	1.268
Nerve invasion	1.687	**0.018**	1.096	2.596
Recurrence-free survival				
Body mass index	0.935	**0.015**	0.886	0.987
Carcinoembryonic antigen	1.125	**0.006**	1.035	1.223
Adjuvant radiotherapy	4.157	**0.000**	2.668	6.477
Nerve invasion	1.866	**0.002**	1.253	2.779

Bold values denote statistical significance.

### 3.3 Discussion

In this retrospective study, we confirmed the safety and effectiveness of the left approach esophagectomy in the perioperative period. Survival analysis suggested the non-inferiority of the left approach regarding OS and RFS despite fewer recurrent laryngeal nerve and thoracic lymph nodes.

Long since the Sweet procedure and its variant (the left cervicothoracic esophagectomy) were invented for esophageal carcinoma (19), there has been debate regarding whether the right or left approach was superior to the maturation of surgical technique and whether an accepted staging system is needed to evaluate procedures systematically. Intriguingly, almost all relevant studies involved Eastern Asian patients (20). On the one hand, the predominant pathological type (ESCC) allows for a diverse approach to radical esophageal resection. On the other hand, the left approach has been an effective technique in the history of thoracic surgery in China. Nevertheless, the debate became heated when a prospective randomized controlled trial was published (8). As Peng et al. suggested, we believe it is too early to conclude the optimal approach for middle and lower ESCC (9). With our experience using both approaches, we believe we can contribute to the debate with the present retrospective PSM study results.

Minimizing the surgical disturbance of the normal physiology process for a procedure that affects up to three body compartments is significant. The left approach, which does not require a change of positioning and re-draping, could minimize the traumatic effect of esophagectomy. Our study showed that, compared to the right approach, the operative time of the left approach was significantly shorter (414.63 ± 130.1 min vs. 488.59 ± 125.93 min, p < 0.001), as was the time to oral intake. For the length of hospital stay, patients who underwent the left approach esophagectomy demonstrated a trend toward superiority. These results agree with current literature (6, 11, 20), confirming the left approach’s potential to limit the invasiveness of esophagectomy.

Nevertheless, criticism was directed at the lymphadenectomy associated with the left approach, not its feasibility or minimal invasiveness. A contemporaneous randomized controlled trial (RCT) suggested that the lymph node yield of the Sweet procedure was inferior to that of the Ivor-Lewis procedure (7); similar results were reached by several retrospective studies (6, 11, 20, 21). Undeniably, the role of lymph node dissection is fundamental in radical resection for ESCC, and there is abundant evidence suggesting that a more extensive lymphadenectomy would, if not promote tumor eradication, at least facilitate accurate staging (22, 23). Some aspects remain worth mentioning before concluding whether the left approach is suitable for middle and lower ESCC resection.

First, the question of whether sufficient lymph nodes could be harvested *via* the left approach is not answered. There is evidence validating the capacity of the lymphadenectomy of the Sweet procedure (3, 24, 25) (and the 4L station lymph nodes), which are clinically significant in ESCC procedures (26). Moreover, the evidence suggests that the modified Sweet procedure can achieve satisfactory upper mediastinal and cervical lymph node dissection (27). The upper mediastinum and base of the neck (which were usually considered the blind spots in the left approach esophagectomy) could be readily exposed; therefore, the yield from these regions is not negligible, though less than from the right approach. As the thoracoscopy could also expose the lymph nodes in the lower mediastinal and abdominal region, an adequate lymphadenectomy could be performed with the left approach. Thus, our study showed no significant difference in the number of lymph nodes harvested between the groups. This finding suggests that the left approach using thoracoscope-assisted surgery and careful handling of the resection margin and the anastomosis achieves a local control rate similar to that of the right approach (3). Similarly, we observed no significant difference in the recurrence rate of the cervical and mediastinal region between the groups in the competing risk analysis. Also, as Xing et al. observed, the performance of lymphadenectomy depended heavily on the surgeon’s operative skill and familiarity with the procedure. Thus, the narrow, “unacceptable” 30 minutes of the Sweet procedure in the RCT of Chen et al. probably indicated relatively insufficient experience, undermining the validity of their results (8, 10). The efficacy of lymphadenectomy of the upper mediastinum region of the right approach deserves recognition; however, the comparison of the capacity of lymphadenectomy of the two approaches awaits more meticulously and systematically designed RCTs.

Although it is clear that better exposure of upper mediastinum is acquired during the right approach procedure, the causal relationship between a more extended lymph node dissection and better outcomes has yet to be established. Akiyama et al. reported that extensive (i.e., three-field lymph node dissection) significantly improved long-term survival compared to less extensive lymphadenectomy (22). Chen et al. also showed that the extended lymphadenectomy benefited lymph node-positive ESCC patients (28). By contrast, there is evidence from an RCT suggesting that the three-field lymphadenectomy (compared to the two-field lymphadenectomy) did not improve the OS or the RFS in esophagectomy for middle and lower esophageal cancer (29). Lagergren et al. investigated the Sweden esophageal cancer population who underwent curative surgery and found that compared to the limited lymphadenectomy (fewer than ten nodes), a more extended lymph node resection (21–52 nodes) did not improve overall and RFS (30). Our study also indicated that a more extensive lymphadenectomy (more than 27 nodes) for node-positive patients improved neither the OS nor RFS. These findings suggest that it is at least reasonable to assume that lymphadenectomy in the radical resection of middle and lower ESCC might control stage migration more than eradicate tumor cells in curative resections of breast cancer, pancreatic cancer malignancies, and tumors of gastric origin. Thus, it is possible that retrieving representative nodes in the upper mediastinal and cervical region is sufficient for the patient without apparently clinically-positive lymph nodes instead of complete dissection.

From a practical point of view, we believe that the Sweet procedure and its cervicothoracic dual-incision variant possess the capacity to achieve adequate lymphadenectomy for middle and lower ECSS, comparable to the right approaches. However, in the clinical scenario where a thorough exposure and dissection of the upper mediastinal lymph node is mandatory (e.g., possible upper mediastinal lymphadenopathy suggested by the preoperative evaluation or when the tumor is expected to be more closely related to the carina or the right main bronchus), the right approach is preferred at our center. This is because the upper mediastinum and other related structures can be reached more efficiently using the right approach. When these mandatory factors are absent, deciding whether to perform the left and right approaches depends on the patient’s status and the surgeon’s preference. With better field exposure and more precise performance, the minimally invasive approach to upper mediastinal and recurrent laryngeal nerve lymph nodes would contribute to adequate lymph node evaluation in the left approach esophagectomy. Therefore, we believe the left approach is valuable for middle and lower ESCC.

This study has several limitations. First, it is better to utilize clinical rather than pathological status to perform stratified analysis. However, without the routinely performed trans-esophageal ultrasound, the degree of precision of preoperative T and N staging is questionable. Hopefully, the issue will be addressed in future studies. Second, although PSM would theoretically minimize the controllable bias of a retrospective designed study, this balance comes at the expense of sample size, and there are confounding factors due to the nonrandomized nature of our research. For example, there is surgeon’s discretion regarding whether to choose the left approach esophagectomy; the decision might derive from preoperative data that are not included in our dataset; finally, PSM cannot control for individual experience. Third, according to current guidelines, the proportion of higher-staged patients who received the neoadjuvant and adjuvant therapies was unsatisfactory. This phenomenon was partially because the survival benefit of neoadjuvant therapy was controversial at the time (31). The non-R0 resection rate (2.34%) was relatively low at our center and was partially derived from the patient’s eagerness to undergo surgery as soon as possible and the fear that chemoradiotherapy would have adverse effects that were once common in mainland China. Before surgery, we routinely explain the pros and cons of neoadjuvant and adjuvant therapies and document the decision-making process. The rate of patient acceptance of neoadjuvant and adjuvant therapy has been rising since then, and we are eager to continue recommending treatment plans that could benefit patients, according to current evidence. Fourth, due to the limits of our database, 38 patients (12.9%) were lost to follow-up. Fortunately, the distribution of follow-up loss was relatively even between the groups (24 in the right group and 14 in the left group). The details of the neoadjuvant and the adjuvant therapy were not included in our database, which could mask underlying discrepancies among patients in different groups and influence the results.

In conclusion, esophagectomy using a qualified lymphadenectomy could be conducted *via* the left approach with similar outcomes to the right approach. The left approach was associated with non-inferior long-term OS and RFS. A minimum number of lymph nodes needed to be resected to ensure better survival in surgically-resected N0 middle and lower ESCC. A well-designed multi-center RCT should be conducted to compare the oncological effects of the two approaches.

## Data availability statement

The raw data supporting the conclusions of this article will be made available by the authors, without undue reservation.

## Ethics statement

The studies involving human participants were reviewed and approved by the ethics committee of Peking University First Hospital. The patients/participants provided their written informed consent to participate in this study.

## Author contributions

JL and GL conceived and designed the study. JWL, WH, KQ, and XZ performed data collection and processing and analysis of the data. XZ wrote the manuscript. All authors contributed to the article and approved the submitted version.
